# Testosterone Deficiency Promotes Arterial Stiffening Independent of Sex Chromosome Complement

**DOI:** 10.21203/rs.3.rs-3370040/v1

**Published:** 2023-10-14

**Authors:** Anil Sakamuri, Bruna Visniauskas, Isabella Kilanowski-Doroh, Alexandra McNally, Ariane Imulinde-Sugi, Anne Kamau, Divya Sengottaian, John McLachlan, Montserrat Anguera, Franck Mauvais-Jarvis, Sarah Lindsey, Benard Ojwang Ogola

**Affiliations:** Augusta University Medical College of Georgia; Tulane University School of Medicine; Tulane University School of Medicine; Tulane University School of Medicine; Tulane University School of Medicine; Augusta University Medical College of Georgia; Augusta University Medical College of Georgia; Tulane University School of Medicine; Penn Vet: University of Pennsylvania School of Veterinary Medicine; Tulane University School of Medicine; Tulane University School of Medicine; Augusta University Medical College of Georgia

**Keywords:** Sex hormones, sex chromosomes, vascular mechanics, pulse wave velocity, arterial stiffening

## Abstract

**Background:**

Testosterone plays a vital role in men’s health. Lower testosterone level is associated with cardiovascular and cardiometabolic diseases, including inflammation, atherosclerosis, and type 2 diabetes. Testosterone replacement is beneficial or neutral to men’s cardiovascular health. Testosterone deficiency is associated with cardiovascular events. Testosterone supplementation to hypogonadal men improves libido, increases muscle strength, and enhances mood. We hypothesized that sex chromosomes (XX and XY) interaction with testosterone plays a role in arterial stiffening.

**Methods:**

We used four core genotype male mice to understand the inherent contribution of sex hormones and sex chromosome complement in arterial stiffening. Age-matched mice were either gonadal intact or castrated for eight weeks, followed by an assessment of blood pressure, pulse wave velocity, echocardiography, and ex vivo passive vascular mechanics.

**Results:**

Arterial stiffening but not blood pressure was more significant in castrated than testes-intact mice independent of sex chromosome complement. Castrated mice showed a leftward shift in stress-strain curves and carotid wall thinning. Sex chromosome complement (XX) in the absence of testosterone increased collagen deposition in the aorta and Kdm6a gene expression.

**Conclusion:**

Testosterone deprivation increases arterial stiffening and vascular wall remodeling. Castration increases Col1α1 in male mice with XX sex chromosome complement. Our study shows decreased aortic contractile genes in castrated mice with XX than XY sex chromosomes.

## Plain language summary

Cardiovascular disease is the leading cause of death worldwide. Cardiovascular disease presents differently in men and women. While men develop plaque buildup in large arteries, women develop buildup in the microvessels in the heart. Arterial stiffening, which is the hardening of arteries, increases with age in both men and women. Aging, coupled with the decline in sex hormones, exacerbates cardiovascular disease in women compared to men. Men with XY sex chromosomes have higher circulating testosterone, while women with XX sex chromosomes have increased circulating estradiol. The potential benefits of sex hormone replacement therapy are shown in men and women. Indeed, testosterone replacement deficiency is associated with adverse cardiovascular outcomes in men. Whether adverse events are dependent or independent of sex hormones’ interaction with sex chromosomes is unknown. This study used the four core genotype mice comprising males with either XX or XY sex chromosome complement. We show castration increases arterial stiffening and collagen deposition on the arterial wall. We also identified the escapee and smooth muscle contractile genes that may play a role in arterial stiffening. Our data suggests that testosterone deprivation mediates arterial stiffening and remodeling.

## Background

Endogenous sex hormones, estradiol in women and testosterone in men, prevent CVD; however, it is debatable whether the benefits are mediated by sex hormones, sex chromosomes, or their interaction [1–3]. Sexual dimorphism in mammals plays a significant role in the development of CVD, including arterial stiffening [4, 5]. Pulse wave velocity (PWV), an indicator of arterial stiffening, is an independent predictor of cardiovascular mortality and events [6]. The Baltimore Longitudinal Study of Aging showed an increase in PWV and blood pressure in men compared to women, widening the sex difference gap [7]. Studies have shown that PWV either precedes or comes after the development of hypertension [8, 9]. Even so, increasing arterial stiffening is associated with cognitive decline, impairment of kidney function, and CVD in a sex-dependent and independent manner.[10–12]

Sex chromosomes (XX and XY) independent of sex hormones show sex differences in females and males [13]. The male Y chromosome is inherited paternally from father to son [13]. The Y chromosome encodes the Sry gene that instructs the formation of male gonads (testes) and spermatogenic pathways [14]. The absence of testes makes cells to differentiate into ovaries, indicated by the oogenesis pathway [14]. Association the Y chromosome with hypertension is shown in the spontaneously hypertensive rat with identification of a Y-linked locus, suggesting a Y chromosome linkage to blood pressure [15, 16]. Recently, it was shown that the hematopoietic loss of the Y chromosome contributes to cardiac dysfunction [17]. Altogether, the contribution of sex chromosomes is equally significant to sex hormones in understanding and delineating sex differences in CVD.

The X chromosome comprises less than five percent of the human genome and plays a significant role in sex differences across various tissues, including the heart, aorta, and adipocytes [18–20]. While females have two X chromosomes, males have one copy [21]. For dosage compensation of X-linked genes, one of the X chromosomes in female cells is randomly selected for inactivation [22]. X chromosome inactivation (XCI) is initiated and maintained by the long non-coding RNA X inactive specific transcript (Xist), along with various heterochromatic histone modifications and DNA methylation [23]. The inactive X chromosome is epigenetically distinct from the active X in female cells [24]. Genes that escape XCI are expressed from the inactive and active X chromosomes [18]. The XCI escapee genes play a crucial role in CVD, including atherosclerosis and fibrosis in valvular interstitial cells [4, 18, 25, 26].

To understand the contribution of sex hormones, sex chromosomes, and their interaction in CVD in males and females, we used the four-core genotype (FCG) mouse model [27, 28]. In this mouse model, Sry gene has been deleted from the Y chromosome and inserted into an autosome, resulting in (XX and XY^−^) female with ovaries and (XX and XY^− Sry^) male with testes [27, 28]. Therefore, the mice can be used to test for the effect of sex hormones, sex chromosomes, and their interaction. In this study, we sought to clarify whether the interaction of sex hormones and sex chromosomes promotes arterial stiffening in male mice.

## Methods

### Animals

Four core genotype (FCG) mice on C57BL/6J background were used. Studies were conducted in mice between 15–16 weeks old. Male (M) mice XY^− Sry^ were obtained from Dr. Franck Mauvais-Jarvis and were mated with female (F) breeders (XX) on C57bl/6J (RRID: IMSR_JAX:000664) background purchased from Jax Labs (ME, USA). Mating XY^− Sry^ and XX mice produced the four-core genotypes, including XXF, XXM, XY^−^F, and XY^− Sry^M. Mice were genotyped with the following primers: Transgene Forward Sry: 5’ AGC CCT ACA GCC ACA TGA TA 3’, Transgene Reverse Sry: 5’ GTC TTG CCT GTA TGT GAT GG 3’, Forward: 5’ CTG GAG CTC TAC AGT GAT GA 3’, Reverse: 5’ CAG TTA CCA ATC AAC ACA TCA C 3’, Internal positive control: 5’ CAA ATG TTG CTT GTC TGG TG 3’ and Internal positive control: 5’ GTC AGT CGA GTG CAC AGT TT 3’. All mice were maintained at Tulane University in a temperature-controlled vivarium under a 12-hour dark and light cycle with free access to standard chow and drinking water. Animal experiments followed the ARRIVE guidelines [29] approved by Augusta and Tulane University Institutional Animal Care and Use Committee.

### Castration

Mice were at eight weeks of age followed by an additional eight weeks to clear endogenous testosterone predominantly from testes. All procedures followed aseptic techniques. Briefly, mice were weighed and placed under a heating pad with 3–4% isoflurane-oxygen mixture, eye cream was applied, and hair was shaved around the incision site. Alcohol (70%) and betadine were used to clean incision sites. This was followed by administration of Buprenorphine 0.1 mg/kg. Castration was performed by locating the scrotum and making a small midline cut above the bladder followed by excision of testicles. Muscle tissue was sutured with absorbable suture and the skin was stapled. Mice were left on heating pads to recover and monitored for pain and stress. Awake and alert mice were placed back in their cages with daily monitoring post-surgical monitoring for wound healing and distress.

### Tail Cuff Plethysmography

Blood pressure (BP) was measured non-invasively using the CODA 4-Channel Blood Pressure System (Kent Scientific, Torrington, CT) in conscious mice as previously described [30]. Three days of acclimation was followed by one week of BP measurements in the morning. Tail temperature and platform was warmed to 30ºC before beginning the inflation protocol, which consisted of 10 cycles of cuff inflation to 250 mmHg followed by a 20 second deflation. Measurements without a definitive inflection point indicating the return of blood flow or with a blood flow volume of less than 30 µl were excluded. The average BP was recorded for each day, and the final BP was reported as the mean of the daily averages after excluding days that were ± 2 standard deviations [30, 31].

### Pulse Wave Velocity and Echocardiography

PWV was performed as previously described by our group [30, 31]. Briefly, Vevo^®^ 1100 ultrasound (VisualSonics, Toronto, ON) was used for cardiovascular analysis. Anesthesia was induced using 3% isoflurane/oxygen mixture and data obtained under 1.5% isoflurane/oxygen mixture. Mice were maintained in supine position on a 37°C heated EKG platform. Shaving cream was applied to the chest, abdomen, and around the throat and wiped with wet gauze. Intracarotid and abdominal pulse wave velocity PWV were measured. Cardiac function was assessed in short axis view in M-mode for left ventricular function.

### Passive Biaxial Mechanical Testing

The carotid artery was dissected and cannulated onto 500µm needle secured with a nylon suture on a pressure myograph system in Hank’s Balanced Salt Solution as previously described [31, 32]. Pressure was applied to the lumen of the vessel and the outer diameter was optically tracked. Biaxial phenotyping was performed as previously described [31, 32]. Pressure-diameter preconditioning was performed from 10 to 150 mmHg. The stretch ratio was assessed by dividing the loaded to unloaded axial length and used to determine the adjusted wall thickness during pressurization. Distensibility was calculated as the percent of starting external diameter. Stress was calculated as (σ) = (P in dyns/cm^2^*D_internal_)/(2*WT), while strain (ε) = D_internal_-D_10mmHg_/D_10mmHg_.

### Quantitative Polymerase Chain Reaction

Mice aorta samples were homogenized in lysis buffer (RLT buffer, Qiagen) using bead homogenizer. The total RNA was isolated using RNeasy plus mini kit (Qiagen, cat. No. 74136). Finally, RNA pellet was resuspended in 25µl of Rnase free water, purity and concentrations were measured by nano spectrometer (Implen Nano photometer, N50). cDNA synthesis was performed with superscript IV Mater mix (cat. No. 11766050). Then real time PCR was carried using PowerTrack SYBR master mix (cat. No A46109) on QuantStudio 3 Real-Time PCR System (Applied Biosystems). Comparative cycle method (2– ^(ΔΔCt)^) was applied for gene expression analysis and β-actin gene was used as an internal control. All the target gene primers were procured from IDT technologies, the IDT predesigned primer IDs were mentioned in the supplementary (Table 1).

### Histology

Aorta samples were fixed in 10% formalin overnight followed by paraffin embedding. Paraffin blocks were serially cut into 5µm sections and stained Masson’s trichrome (MTC: Blue-Collagen; Red-Muscle) or Van Gieson’s stain (Black-elastin bands). Immunofluorescence staining was done using Col1a1(CST#72026), Alpha-Smooth Muscle Actin Monoclonal Antibody 1A4 (RRID: AB_557419), and DAPI (ThermoFisher, D1306). Fluorescent anti-rabbit (RRID: AB_143165) and anti-mouse (RRID: AB_2536180) Images were taken using EVOS cell imaging system. Analysis was done using ImageJ software expressed as percent of total area fraction.

### Transmission Electron Microscopy

Testicles from XYM and XXM mice were fixed in paraformaldehyde and glutaraldehyde in 0.1 M sodium cacodylate (NaCac) buffer, pH 7.4. Postfixing was performed in osmium tetroxide in NaCac, stained in a block with uranyl acetate, and dehydration was performed with ethanol series, and embedded in Epon-Araldite resin. The block was trimmed to permit proper orientation of the testicle during imaging. Diamond knife was used to cut sections on a Leica EM UC7 ultramicrotome (Leica Microsystems, Inc, Bannockburn, IL), collected on copper grids, and uranyl acetate and lead citrate was used for staining. JEM 1400 flash transmission electron microscope (JEOL USA Inc., Peabody, MA) fitted with a CMOS CCD camera was used for imaging.

### Statistics

Data was analyzed using GraphPad Prism version 9.1 (GraphPad Software, San Diego, CA). Outliers were identified by the ROUT method (Q = 1%). 2-way ANOVA was used to compute the main effect, while Sidak’s and uncorrected Fisher’s Least Significant Difference multiple comparisons test were used to determine the difference between groups. The unpaired t-test was used to compare the area under the curve difference between the two groups. All data are presented as means ± S.E.M, and P < 0.05 was considered significant.

## Results

### Castration increases pulse wave velocity independent of blood pressure.

**Figure 1:**
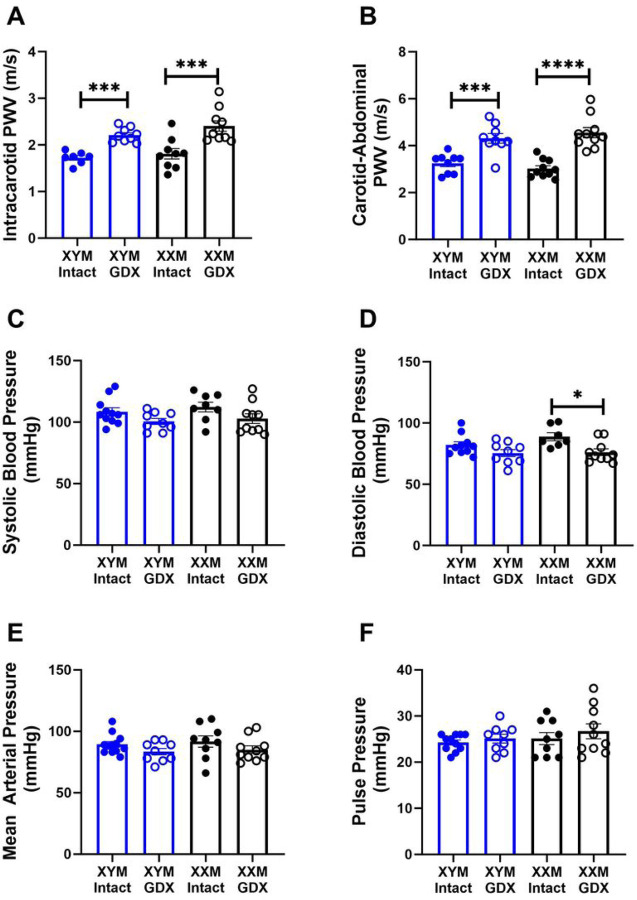
Increased arterial stiffness is not coupled with blood pressure change in gonadectomized mice. A. Intracarotid PWV was significantly higher in gonadectomized than gonadal intact mice in XYM and XXM (F (1, 33) =23.9) ***P<0.001). Similarly, B. Carotid-Abdominal PWV was higher in gonadectomized than gonadal intact mice in XYM and XXM; (F (1, 36) =35.9) ***P<0.001). Blood pressures including C. Systolic (F (1, 34) =6.1) P=0.02) and D. Diastolic (F (1, 32) =10.9) P=0.002) were significantly different but not E. Mean Arterial (F (1, 36) =3.9) P=0.06) and F. Pulse (F (1, 35) =1.1) P=0.002). All data sets were computed as mean fold change ± SEM and 2-Way ANOVA was used to compute column effect, row effect and their interaction. Post hoc Sidak’s multiple comparisons test was used. *P<0.05, **P<0.01, ***P<0.001 and ****P<0.0001 were considered significant.

Intracarotid PWV was higher in castrated than testes intact mice (Fig. 1A; P < 0.0001). Post hoc comparisons indicated increased arterial stiffening in testes intact vs. gonadectomized (GDX) mice in (XYM: 1.7 ± 0.1 vs. 2.2 ± 0.1 m/s; P = 0.002) and (XXM: 1.8 ± 0.1 vs. 2.4 ± 0.1 m/s; P < 0.0001). Similarly, carotid to abdominal PWV indicated a significant castration effect (Fig. 1B; P < 0.0001). Multiple comparisons tests showed increased PWV in testes intact vs. GDX mice in (XYM: 3.2 ± 0.1 vs. 4.3 ± 0.2 m/s; P = 0.001) and (XXM: 3.0 ± 0.1 vs. 4.6 ± 0.2 m/s; P < 0.001). There was no significant main effect of castration on systolic blood pressure (Fig. 1C) in XYM (108 ± 3 vs. 100 ± 2 mmHg, t = 1.7, DF = 34; P = 0.2) and XXM (112 ± 4 vs. 103 ± 4, t = 1.8, DF = 34; P = 0.1) mice. However, diastolic blood pressure (Fig. 1D) showed significant castration effect in XXM (89 ± 3 vs. 76 ± 3, t = 3, DF = 32; P = 0.01) but not in XYM (82 ± 2 vs. 75 ± 3 mmHg, t = 1.7, DF = 32; P = 0.2) mice. Mean arterial pressure (Fig. 1E) was not significantly different in castrated versus testes intact XYM (89 ± 2 vs. 83 ± 3 mmHg, t = 1.4, DF = 36; P = 0.3) and XXM (91 ± 5 vs. 85 ± 3, t = 1.4, DF = 36; P = 0.3) mice. We also calculated the difference in systolic and diastolic pressures shown by pulse pressure (Fig. 1F) that was not significantly different in XYM (24 ± 1 vs. 25 ± 1 mmHg, t = 0.8, DF = 35; P = 0.3) and XXM (25 ± 1 vs. 27 ± 2, t = 0.5, DF = 35; P = 0.3) mice.

### Castration reduces distensibility in XXM mice at higher pressures.

**Figure 2:**
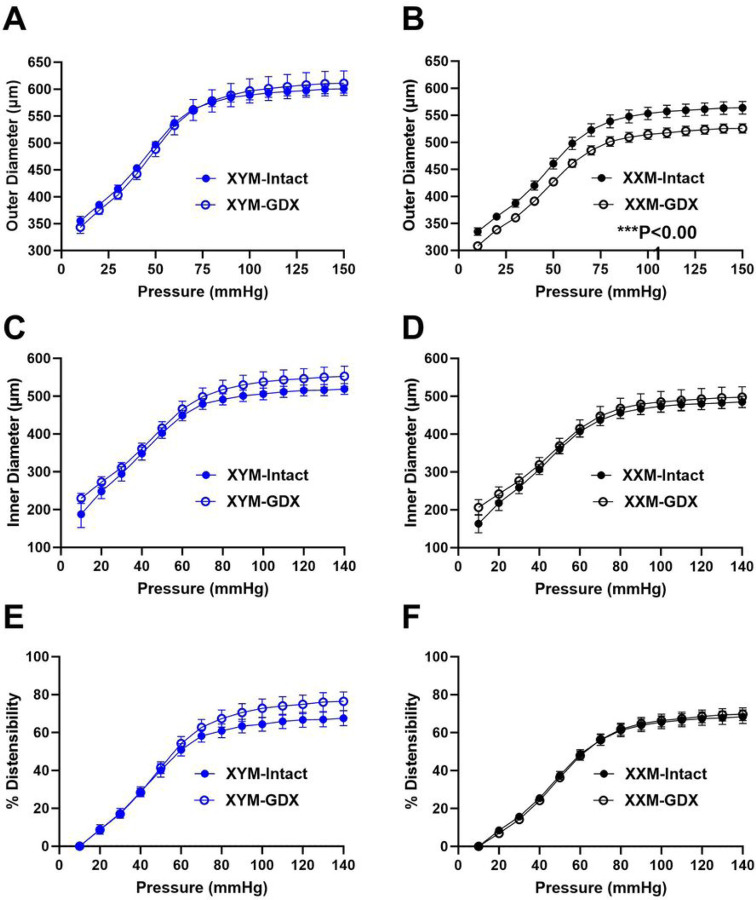
Passive pressure-diameter relationship and distensibility of carotid arteries. The pressure-outer diameter of **A.** XYM was not significant (t=0.02, df=17; P=0.9) and **B.** XXM gonadal intact is greater than GDX (t=4, df=15; P=0.0003). Pressure-inner diameters were not significantly different between castrated and intact mice in **C.** XYM (t=1.4, df=13; P=0.2) and **D.** XXM (t=0.8, df=15; P=0.4). **E.** Distensibility was not significant in either XYM (t=1.7, df=15; P=0.1) and **F.** XXM mice (t=1.2, df=17; P=0.8). An unpaired t-test was used to compare gonadal intact and GDX mice AUC.

We performed a passive pressure myograph on carotid arteries to determine whether PWV alters biaxial vascular parameters assessed ex vivo. Pressure-outer diameter relationship area under the curve (AUC) was not different between testes intact vs. GDX XYM (Fig. 2A: 7639 ± 569 vs. 74667 ± 1482; P = 0.9); however, gonadectomized XXM compared to testes-intact XXM mice showed decrease in outer diameter AUC (Fig. 2B: 69808 ± 842 vs. 64921 ± 586; P < 0.001). Pressure-inner diameter AUC relationship was not significantly different in castrated versus testes-intact XYM (Fig. 2C: 56159 ± 1052 vs. 59409 ± 1706; P = 0.01) and XXM (Fig. 2D: 51512 ± 1202 vs. 53318 ± 1979; P > 0.9) mice. To test for the elastic property of the arterial wall, we observed greater distensibility versus pressure in castrated than testes intact XYM (Fig. 2E: 6254 ± 211 vs. 6861 ± 302; P = 0.1) but not in XXM (Fig. 2F: 6181 ± 214 vs. 6189 ± 219; P > 0.9) mice.

### Gonadectomy decreases compliance and shifts stress-strain curves to the left.

**Figure 3:**
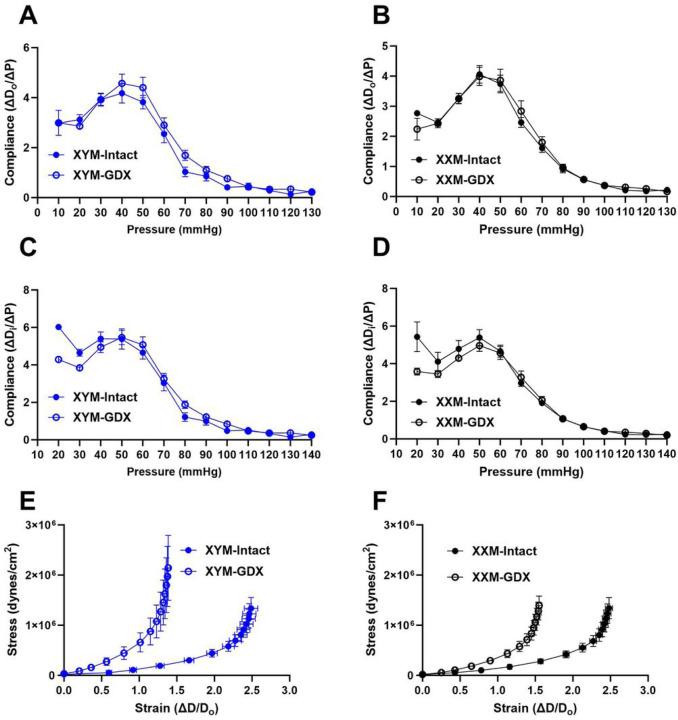
adectomy decreases compliance and shifts stress-strain curves to the left. **Gon** Compliance calculated form outer diameter was not different in castrated versus gonadal intact **A.** XYM (t=1.2, df=12; P=0.3) and **B**. XXM (t=0.3, df=17; P=0.8) mice. Using inner diameter dimensions also did not indicate significant difference in compliance in **C.** XYM (t=0.02, df=12; P=0.9) and **D.** XXM (t=0.7, df=17; P=0.5) mice. Gonadectomy shifted stress-strain curves to the left in **C.** XYM (t=0.07, df=12; P=0.9) and **D.** XXM (t=1.9, df=17; P=0.08) mice. An unpaired t-test was used to compare gonadal intact and GDX mice AUC.

Given the difference in pressure diameter and distensibility, we examined whether there were changes in compliance, a material property impacted by pressure and stiffness. Computing compliance using outer diameter changes per pressure showed no significant differences in testes intact and castrated XYM (Fig. 3A: 250 ± 16 vs. 224 ± 13; P = 0.3) and XXM (Fig. 3B: 218 ± 16 vs. 213 ± 11; P = 0.8) mice. Similarly, compliance calculated by inner diameter showed no significant difference in XYM (Fig. 3C: 300 ± 15 vs. 299 ± 16; P > 0.9) and XXM (Fig. 3D: 272 ± 16 vs. 293 ± 22; P = 0.5) in testes intact and castrated mice. Increased PWV with castration, shown in Fig. 1, prompted us to calculate the circumferential stress-strain of carotid arteries. Our data indicated increased stiffening shown with a leftward shift of stress-strain curves in GDX XYM (Fig. 3E: 675190 ± 70665 vs. 687679 ± 134296; P = 0.9) and GDX XXM (Fig. 3F: 692280 ± 81519 vs. 498251 ± 66164; P = 0.09) than testes intact mice.

### Castration-induced carotid wall thinning and aortic collagen deposition.

**Figure 4:**
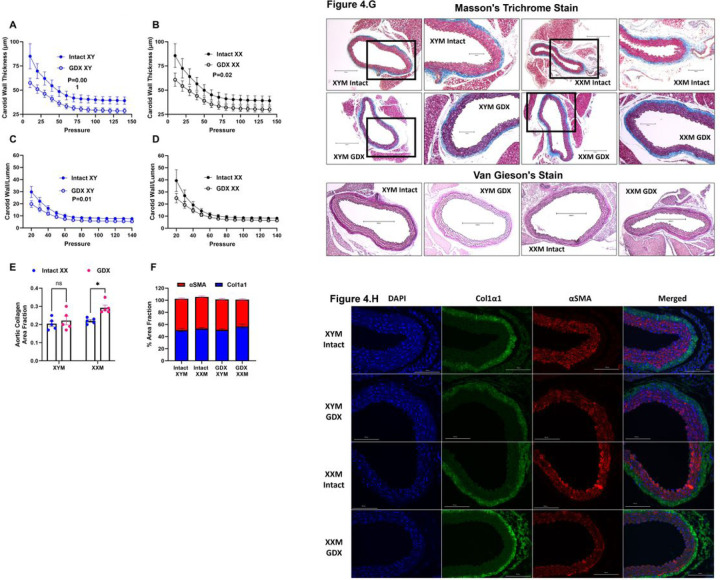
Castration-induced carotid wall thinning and aortic collagen deposition. Castration decreased carotid wall thickness in **A.** XYM (t=4, df=13; P=0.001) and **B.** XXM (t=3, df=17; P=0.02) mice. Carotid wall to lumen ration was significantly different in **C.** XYM (t=3, df=17; P=0.009), but not **D.** XXM (t=2, df=12; P>0.09). **E.** Gonadectomy significantly increased collagen area fraction XXM (t=3, df=16; P=0.02), but not XYM (t=0.8, df=16; P=0.8) mice. **F.** Aortic α smooth muscle was not significantly impacted by castration (P=0.5), however, col1a1 was increased in gonadectomized XX mice (t=4.0, df=16; P=0.002). Representative images of Masson’s trichrome staining (Figure 4G) and Immunofluorescence (Figure 4H).

We assessed carotid wall thickness and wall-to-lumen ratio to determine whether testosterone deprivation mediated geometrical changes on the arterial wall. Our data uncovered the wall thinning effect due to castration versus testes intact XYM (Fig. 4A: 6228 ± 337 vs. 4586 ± 234; P = 0.001) and XXM (Fig. 4B: 6327 ± 483 vs. 4860 ± 304; P = 0.02) mice. Wall to lumen ratio was significantly different in castrated versus testes intact XYM (Fig. 4C: 1362 ± 103 vs. 972 ± 84; P = 0.01) but not XXM (Fig. 4D: 1577 ± 199 vs. 1184 ± 117; P = 0.09) mice. Whether androgen deprivation plays a role in aortic wall remodeling is unknown; therefore, we assessed collagen deposition on the aorta using Masson’s Trichrome (Blue; Fig. 4G) staining and found a significant collagen increase in castrated compared to testes intact XXM (Fig. 4E: 0.2 ± 0.01 vs. 0.02; P = 0.02) but no difference in XYM mice. Representative images (Fig. 4G) show MTC staining in the top two rows and VVG staining (no elastin strand breaks; black strands) in the bottom row.

To determine which collagen specifically is increasingly deposited, we performed immunofluorescence (Fig. 4H), showing Col1a1 stained green was increased by castration (Fig. 4F and 4H; P = 0.004) and shown to be higher in gonadectomized than testes intact XXM mice (Fig. 4F and 4H; P = 0.002) but no significant difference in XYM aortic cross-sections. Smooth muscle α-actin stained red (Fig. 4F and 4H; P = 0.5) used as a counterstain was decreased in castrated XXM mice, but no statistical significance was detected. Representative immunofluorescence staining for αSMA and Col1a1 is shown in (Fig. 4H).

### Gonadectomy downregulates contractile genes in the aorta of XX mice.

**Figure 5:**
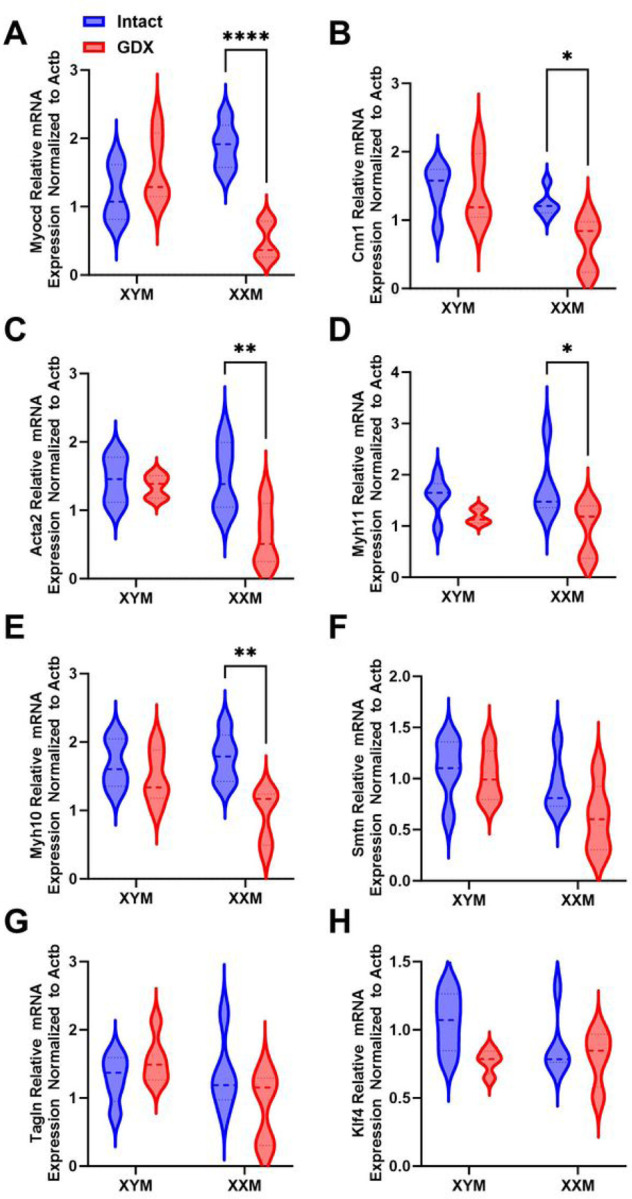
Mice with XX sex chromosome complement had decrease in SMC contractile genes after gonadectomy. **A.** Myocd (t=6, df=16; P<0.0001), **B.** Cnn1 (t=2, df=16; P=0.03), **C.** Acta2 (t=3, df=14; P=0.007), **D.** Myh11 (t=3, df=16; P=0.01), and **E.** Myh10 (t=4, df=16; P=0.003), mRNA was decreased in gonadectomized XXM mice. However, there was no significant castration effect in **F.** Smtn (F (1, 16)=2.4); P=0.1), **G.** Tagln (F (1, 16)=0.2); P=0.6), and **H.** Klf4 (F (1, 14)=3.9); P=0.07). All data sets were computed as mean fold change ± SEM and 2-Way ANOVA was used to compute column effect, row effect and their interaction. Uncorrected Fisher’s LSD was used for multiple comparisons *P<0.05, **P<0.01, and ****P<0.0001.

Collagen deposition on the aorta can modulate smooth muscle functional response; therefore, we measured contractile gene expression in testes intact and gonadectomized mice. Castration significantly decreased contractile genes in XXM but not XYM mice, including Myocardin (Fig. 5A: Myocd; P < 0.0001), Calponin (Fig. 5B: Cnn1; P = 0.03), Smooth muscle cell alpha-actin (Fig. 5C: Acta2; P = 0.006), Myosin heavy chain 11 (Fig. 5D: Myh11; P = 0.01) and (Fig. 5E: Myh10; P = 0.002). There were no significant changes in Smoothelin (Fig. 5F; Smtn), Transgelin (Fig. 5G; Tagln) and Kruppel-like factor 4 (Fig. 5H; KLF4)

### Castration increases lysine demethylase 6a gene expression.

**Figure 6:**
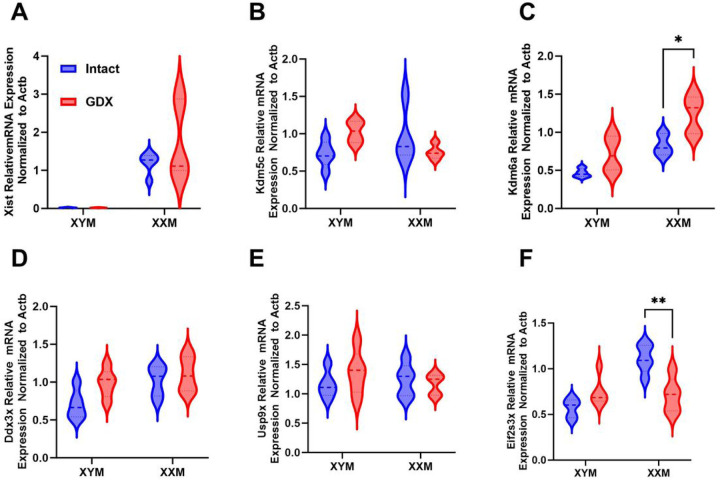
Impact of castration on X link gene expression in mice. **A.** Distinctive Xist mRNA expression in XX than XY mice (F (1, 15) =34); P<0.0001). **B.** Kdm5c expression was not impacted by gonadectomy in either XXM or XYM mice (F (1, 13) =0.2) P=0.7). **C.** Kdm6a expression was significantly higher in castrated XXM mice (t=3, df=12; P=0.01) but not XYM (t=2, df=12; P=0.1). No significant effect of castration as indicated in **D.** Ddx3x (F (1, 16)=4); P=0.06) and **E.** Usp9x (F (1, 16)=0.3) P=0.6). However, **F**. Eif2s3x gene expression was decreased in gonadectomized XXM (t=4, df=16; P=0.02), but not XYM (t=2, df=16; P=0.2) mice. All data sets were computed as mean fold change ± SEM and 2-Way ANOVA was used to compute column effect, row effect and their interaction with Sidak’s multiple comparisons. *P<0.05 and **P<0.01.

The decrease in contractile gene expression in GDX XXM but not GDX XYM compared to testes intact mice prompted us to assess X-linked escapee genes. First, we assessed Xist gene expression, distinguishing XX from XY samples. The data shows a significant (Fig. 6A; P < 0.0001) increase of Xist in GDX and testes of intact XXM but not XYM mice. Lysine demethylase 5c (Fig. 6B; Kdm5c) was not significantly different between groups. However, an increase in Kdm6a was observed in gonadectomized versus testes intact XX mice (Fig. 6C; P = 0.01). Additional escapee genes assessed, including DEAD-Box Helicase 3 X-linked (Ddx3x) and Ubiquitin Specific Peptidase 9 X-linked (Usp9x), were not significantly different between groups (Fig. 6D and Fig. 6E). Eukaryotic translation initiation factor 2 subunit 3, X-linked (Eif2s3x) indicated decreased expression in castrated XXM versus testes intact mice (Fig. 6F; P = 0.002).

### Anabolic effect of testosterone on tissue weights

Given the changes in vascular biomechanics, we assessed whether tissue weights were impacted by castration. Total body weight was decreased in gonadectomized XYM (Supplementary Fig. 1A: 23 ± 0.6 vs. 21 ± 0.3; t = 3, df = 31; P = 0.02) and XXM (Supplementary Fig. 1A: 25 ± 0.7 vs. 20 ± 0.5; t = 7, df = 31; P < 0.0001) compared to testes intact mice. Similarly, kidney/body weight was decreased in castrated XYM (Supplementary Fig. 1B: 6.3 ± 0.1 vs. 5.4 ± 0.2; t = 3, df = 32; P = 0.02) and XXM (Supplementary Fig. 1B: 6.8 ± 0.3 vs. 5.5 ± 0.1; t = 5, df = 32; P < 0.0001) compared to testes intact mice. Whole heart/body weight was not significantly different in castrated and testes intact XYM (Supplementary Fig. 1C: 5.3 ± 0.2 vs. 5.5 ± 0.4; t = 0.5, df = 31; P = 0.9) and XXM (Supplementary Fig. 1C: 5.6 ± 0.4 vs. 5.2 ± 0.2; t = 1, df = 31; P = 0.5) mice. However, echocardiography indicated that castration decreased in left ventricular mass in XYM and XXM compared to testes intact mice (Supplementary Table 2; 120 ± 11 vs. 98 ± 10 mg; P < 0.01 and 125 ± 6 vs. 95 ± 9 mg; P < 0.01). Testicular weight was significantly smaller in XXM compared to XYM mice (Supplementary Fig. 1D: 1.5 ± 0.2 vs. 4.6 ± 0.2; P < 0.0001). Castration significantly decreased seminal vesicle/body weight in XYM (Fig. 7E: 9.0 ± 0.7 vs. 0.6 ± 0.1; P < 0.0001) and XXM (Supplementary Fig. 1E: 9.8 ± 0.5 vs. 0.4 ± 0.1; P < 0.0001) compared to testes intact mice.

Further evaluation of testicular morphology using a transmission electron microscope revealed structural disorganization in XXM than XYM mice. Leydig cells (Supplementary Fig. 2A; White arrow) were abundant and spread out in XYM than XXM sections. Lipid droplets (Supplementary Fig. 2B; Red arrow) were dense in XXM and scarcely distributed in the XYM section. Endoplasmic reticulum (ER) appeared to be organized and interconnected in XYM; however, XXM mice indicated swollen ER with cisternae disorganization (Supplementary Fig. 2C and D; Red asterisk). Mitochondrial morphology in XYM was elongated and numerous in XYM, but XXM showed swollen circular-like mitochondria (Supplementary Fig. 2C and D; Dollar sign).

## Discussion

We report for the first time that castration induces arterial stiffening independent of sex chromosome complement without significantly changing the blood pressure of male four-core genotype (FCG) mice. We show carotid wall thinning and an overall leftward shift of stress-strain curves upon testosterone deficiency, indicating arterial stiffening. The present study shows arterial structural remodeling with increased collagen deposition, including Col1a1, without elastin strand breaks. Due to testosterone deficiency, a substantial decrease in smooth muscle contractile gene expression and an increase in the X-linked escapee gene Kdm6a is indicated in XXM but not XYM mice. Additionally, we show a structural disorganization of testes in XXM than in XYM mice. The results demonstrate the role of sex chromosomes (XX and XY) and sex hormones (testosterone) in vascular biomechanics.

Aging results in a decline of sex hormones, parallel to an increase in arterial stiffening [7]. The use of androgen deprivation therapy and castration in prostate cancer increases arterial stiffening [33]. Furthermore, testosterone deficiency is associated with endothelial dysfunction, demonstrating the essential role of testosterone in vascular health [34]. Study using FCG mice on MF1 background showed no significant difference in blood pressure in XX or XY mice before Angiotensin II infusion recapitulates our findings, indicating lower but not significant systolic blood pressure after gonadectomy [35]. Besides blood pressure, PWV was higher in testosterone-deficient men without CVD, suggesting a protective role for testosterone [36]. It is shown that hypogonadal males have significantly benefited from testosterone therapy to improve libido and mood [3]. Studies have shown that gonadectomy unmasked effects of sex chromosome (XX) in hypertension, atherosclerosis, and ischemia-reperfusion injury in the heart [20, 35, 37]. We showed increased Col1a1 and Kdm6a levels in castrated XX mice that is analogous to a study showing increased adiposity with Kdm5c overexpression, suggesting that the lysine demethylase family of proteins plays an essential role in autosomal gene regulation [19]. However, we did not determine the mechanisms of transcriptional activation of Col1a1 by Kdm6a.

Genes that escape X-chromosome inactivation impact CVD, including atherosclerosis and fibrosis in valvular interstitial cells [4, 18, 25, 26]. We show that Kdm6a plays a role in arterial remodeling and is also implicated in autoimmunity [38]. Additionally, Kdm5c is involved in adiposity, and recently discovered escapee genes Bms and Stx regulate aortic stenosis progression [19, 25]. Kdm6a is associated with adiposity of male and female mice with XX sex chromosome complement [19, 39]. Clinically, men born with an extra X chromosome (Klinefelter syndrome) have an increased incidence of CVD that is not reversed by testosterone replacement [39, 40]. Given that men with Klinefelter syndrome have XXY sex chromosomes, lysine demethylases may be a druggable target to mitigate CVD, including arterial stiffening and metabolic disorders [40–42]. Arguably, Klinefelter individuals have a Y chromosome linked to the Sry gene unlike our mouse model (XXM) with an autosomal Sry gene [43]. However, the Y chromosome, in comparison to the X, encodes very few genes; even so, hematopoietic mosaic loss of the Y chromosome increases CVD risk in men [17].

Extracellular matrix, including collagen deposition and fiber arrangement on the arterial wall, has long been attributed to arterial stiffening and remodeling [30, 31]. In this study, gonadectomy increased collagen deposition in mice with XX sex chromosome suggesting a role for X-linked genes in driving arterial wall remodeling [20]. Our data support a role for Col1a1 in arterial stiffening and remodeling, which aligns with a study in humans and mice showing polymorphism on COL1α1 Sp1 binding site impacts arterial stiffening [44, 45]. Arterial stiffening is coupled with changes in vascular cells; we show a decrease in Myocd, a master regulator of SMC contractile phenotype changes in a similar trend to Acta2, Calponin, and Myh10, and Myh11 [46]. Compelling evidence implicate SMC phenotype switching promotes aortic diseases and our study shows how sex hormones perturbation in mice with XX sex chromosomes is impacted more [47, 48].

The arterial geometry, including wall thickness, contributes to vascular distensibility, incremental elastic modulus, and PWV [45, 49]. Our data shows that carotid walls got thinner while the aorta wall had an increase in collagen deposition, suggesting higher mechanical wall stress associated with aging and hypertension [45, 50]. Stress-strain curves indicated a leftward shift in all groups after gonadectomy recapitulating PWV assessed in vivo [9, 31]. The leftward curve shift after gonadectomy primes the vessels to respond to arterial remodeling, predisposing the vessel to impaired flow-mediated dilation, hypertension, atherosclerosis, and abdominal aortic aneurysms [20, 35, 51].

## Perspectives and significance

While the FCG mouse is a widely considered model for investigating the role of sex hormones, sex chromosomes, and their interaction, the present study has limitations [28]. Unlike wild-type male mice, the FCG male (XYM) model has a Y chromosome independent of the Sry gene that resides in chromosome 3; therefore, we made a general assumption that the chromosomal mutation and Sry transgene location did not skew the cardiovascular phenotype [28]. An alternative approach may involve comparing male mice with a Y-chromosome mutation and a Sry transgene (XY^− Sry^) with a wild-type male mouse comprising a Y chromosome linked to Sry (XY^Sry^). Our study did not assess active vascular properties, including vasoreactivity in smaller arteries shown to impact angiotensin-induced vasodilation involving the type 2 receptor [52]. The Traverse study (NCT03518034) recently showed no adverse effect of testosterone replacement in hypogonadal men, which paves the way for novel testosterone therapies in CVD [53]. Given that the presence of two X chromosomes and the Sry gene impact on CVD is rarely studied, [40] future studies can utilize the mouse XY* model that allows for the generation of mice with XXY sex chromosomes present in Klinefelter men to understand the role of sex chromosomes in vascular disease [43].

## Supplementary Material

Supplement 1

## Highlights

- Castration increases pulse wave velocity independent of blood pressure in male FCG mice.
- Castration-mediated carotid artery leftward shift in stress-strain.
- Arterial remodeling, including collagen deposition and decrease in contractile genes, was more significant in mice with XX sex chromosome complement.

## Editorial Note

17, August, 2026: The funding information listed in the preprint has been corrected in the final published version of record.

## References

[1] Arnold AP, Cassis LA, Eghbali M, Reue K, Sandberg K. Sex Hormones and Sex Chromosomes Cause Sex Differences in the Development of Cardiovascular Diseases. Arterioscler Thromb Vasc Biol. 2017;37:5:746–56; doi:10.1161/ATVBAHA.116.307301. 28279969 PMC5437981

[2] Fernandez-Balsells MM, Murad MH, Lane M, Lampropulos JF, Albuquerque F, Mullan RJ, Clinical review 1: Adverse effects of testosterone therapy in adult men: a systematic review and meta-analysis. J Clin Endocrinol Metab. 2010;95:6:2560–75; doi:10.1210/jc.2009-2575. 20525906

[3] Cunningham GR, Stephens-Shields AJ, Rosen RC, Wang C, Bhasin S, Matsumoto AM, Testosterone Treatment and Sexual Function in Older Men With Low Testosterone Levels. J Clin Endocrinol Metab. 2016;101:8:3096–104; doi:10.1210/jc.2016-1645. 27355400 PMC4971331

[4] Reue K, Wiese CB. Illuminating the Mechanisms Underlying Sex Differences in Cardiovascular Disease. Circ Res. 2022;130:12:1747–62; doi:10.1161/CIRCRESAHA.122.320259. 35679362 PMC9202078

[5] Ogola BO, Zimmerman MA, Clark GL, Abshire CM, Gentry KM, Miller KS, New insights into arterial stiffening: does sex matter? Am J Physiol Heart Circ Physiol. 2018;315:5:H1073–H87; doi:10.1152/ajpheart.00132.2018. 30028199 PMC6415742

[6] Hametner B, Wassertheurer S, Mayer CC, Danninger K, Binder RK, Weber T. Aortic Pulse Wave Velocity Predicts Cardiovascular Events and Mortality in Patients Undergoing Coronary Angiography: A Comparison of Invasive Measurements and Noninvasive Estimates. Hypertension. 2021;77:2:571–81; doi:10.1161/HYPERTENSIONAHA.120.15336. 33390046

[7] AlGhatrif M, Strait JB, Morrell CH, Canepa M, Wright J, Elango P, Longitudinal trajectories of arterial stiffness and the role of blood pressure: the Baltimore Longitudinal Study of Aging. Hypertension. 2013;62:5:934–41; doi:10.1161/HYPERTENSIONAHA.113.01445. 24001897 PMC3880832

[8] Mitchell GF. Arterial stiffness and hypertension. Hypertension. 2014;64:1:13–8; doi:10.1161/HYPERTENSIONAHA.114.00921. 24752432 PMC4063409

[9] Weisbrod RM, Shiang T, Al Sayah L, Fry JL, Bajpai S, Reinhart-King CA, Arterial stiffening precedes systolic hypertension in diet-induced obesity. Hypertension. 2013;62:6:1105–10; doi:10.1161/HYPERTENSIONAHA.113.01744. 24060894 PMC3951434

[10] Dao E, Barha CK, Santos M, Welch M, Liu-Ambrose T. Sex Differences in the Relationship Between Arterial Stiffness and Cognitive Function in Older Adults. J Stroke Cerebrovasc Dis. 2022;31:1:106175; doi:10.1016/j.jstrokecerebrovasdis.2021.106175. 34715522

[11] Townsend RR, Anderson AH, Chirinos JA, Feldman HI, Grunwald JE, Nessel L, Association of Pulse Wave Velocity With Chronic Kidney Disease Progression and Mortality: Findings From the CRIC Study (Chronic Renal Insufficiency Cohort). Hypertension. 2018;71:6:1101–7; doi:10.1161/HYPERTENSIONAHA.117.10648. 29712736 PMC6342478

[12] Mitchell GF, Hwang SJ, Vasan RS, Larson MG, Pencina MJ, Hamburg NM, Arterial stiffness and cardiovascular events: the Framingham Heart Study. Circulation. 2010;121:4:505–11; doi:10.1161/CIRCULATIONAHA.109.886655. 20083680 PMC2836717

[13] Arnold AP. A general theory of sexual differentiation. J Neurosci Res. 2017;95:1–2:291–300; doi:10.1002/jnr.23884. 27870435 PMC5369239

[14] Koopman P. The delicate balance between male and female sex determining pathways: potential for disruption of early steps in sexual development. Int J Androl. 2010;33:2:252–8; doi:10.1111/j.1365-2605.2009.01001.x. 19845801

[15] Turner ME, Farkas J, Dunmire J, Ely D, Milsted A. Which Sry locus is the hypertensive Y chromosome locus? Hypertension. 2009;53:2:430–5; doi:10.1161/HYPERTENSIONAHA.108.124131. 19075093

[16] Ely DL, Turner ME. Hypertension in the spontaneously hypertensive rat is linked to the Y chromosome. Hypertension. 1990;16:3:277–81; doi:10.1161/01.hyp.16.3.277. 2394486

[17] Sano S, Horitani K, Ogawa H, Halvardson J, Chavkin NW, Wang Y, Hematopoietic loss of Y chromosome leads to cardiac fibrosis and heart failure mortality. Science. 2022;377:6603:292–7; doi:10.1126/science.abn3100. 35857592 PMC9437978

[18] Tukiainen T, Villani AC, Yen A, Rivas MA, Marshall JL, Satija R, Landscape of X chromosome inactivation across human tissues. Nature. 2017;550:7675:244–8; doi:10.1038/nature24265. 29022598 PMC5685192

[19] Link JC, Wiese CB, Chen X, Avetisyan R, Ronquillo E, Ma F, X chromosome dosage of histone demethylase KDM5C determines sex differences in adiposity. J Clin Invest. 2020;130:11:5688–702; doi:10.1172/JCI140223. 32701509 PMC7598065

[20] AlSiraj Y, Chen X, Thatcher SE, Temel RE, Cai L, Blalock E, XX sex chromosome complement promotes atherosclerosis in mice. Nat Commun. 2019;10:1:2631; doi:10.1038/s41467-019-10462-z. 31201301 PMC6643208

[21] Maan AA, Eales J, Akbarov A, Rowland J, Xu X, Jobling MA, The Y chromosome: a blueprint for men’s health? Eur J Hum Genet. 2017;25:11:1181–8; doi:10.1038/ejhg.2017.128. 28853720 PMC5643963

[22] Lyon MF. Gene action in the X-chromosome of the mouse (Mus musculus L.). Nature. 1961;190:372–3; doi:10.1038/190372a0. 13764598

[23] Yin H, Wei C, Lee JT. Revisiting the consequences of deleting the X inactivation center. Proc Natl Acad Sci U S A. 2021;118:25; doi:10.1073/pnas.2102683118. PMC8237661 34161282

[24] Pyfrom S, Paneru B, Knox JJ, Cancro MP, Posso S, Buckner JH, The dynamic epigenetic regulation of the inactive X chromosome in healthy human B cells is dysregulated in lupus patients. Proc Natl Acad Sci U S A. 2021;118:24; doi:10.1073/pnas.2024624118. PMC8214693 34103397

[25] Aguado BA, Walker CJ, Grim JC, Schroeder ME, Batan D, Vogt BJ, Genes That Escape X Chromosome Inactivation Modulate Sex Differences in Valve Myofibroblasts. Circulation. 2022;145:7:513–30; doi:10.1161/CIRCULATIONAHA.121.054108. 35000411 PMC8844107

[26] Buono MF, Benavente ED, Daniels M, Mol BM, Mekke JM, de Borst GJ, X chromosome inactivation skewing is common in advanced carotid atherosclerotic lesions in females and predicts secondary peripheral artery events. Biol Sex Differ. 2023;14:1:43; doi:10.1186/s13293-023-00527-6. 37408072 PMC10324263

[27] Lovell-Badge R, Robertson E. XY female mice resulting from a heritable mutation in the primary testis-determining gene, Tdy. Development. 1990;109:3:635–46; doi:10.1242/dev.109.3.635. 2401216

[28] Burgoyne PS, Arnold AP. A primer on the use of mouse models for identifying direct sex chromosome effects that cause sex differences in non-gonadal tissues. Biol Sex Differ. 2016;7:68; doi:10.1186/s13293-016-0115-5. 27999654 PMC5154145

[29] Percie du Sert N, Ahluwalia A, Alam S, Avey MT, Baker M, Browne WJ, Reporting animal research: Explanation and elaboration for the ARRIVE guidelines 2.0. PLoS Biol. 2020;18:7:e3000411; doi:10.1371/journal.pbio.3000411. 32663221 PMC7360025

[30] Ogola BO, Abshire CM, Visniauskas B, Kiley JX, Horton AC, Clark GL, Sex Differences in Vascular Aging and Impact of GPER Deletion. Am J Physiol Heart Circ Physiol. 2022; doi:10.1152/ajpheart.00238.2022. PMC9306784 35749718

[31] Ogola BO, Clark GL, Abshire CM, Harris NR, Gentry KL, Gunda SS, Sex and the G Protein-Coupled Estrogen Receptor Impact Vascular Stiffness. Hypertension. 2021;78:1:e1–e14; doi:10.1161/HYPERTENSIONAHA.120.16915. 34024124 PMC8192475

[32] Ogola BO, Abshire CM, Visniauskas B, Kiley JX, Horton AC, Clark-Patterson GL, Sex differences in vascular aging and impact of GPER deletion. Am J Physiol Heart Circ Physiol. 2022;323:2:H336–H49; doi:10.1152/ajpheart.00238.2022. 35749718 PMC9306784

[33] Ng CF, Chiu PKF, Yee CH, Lau BSY, Leung SCH, Teoh JYC. Effect of androgen deprivation therapy on cardiovascular function in Chinese patients with advanced prostate cancer: a prospective cohort study. Sci Rep. 2020;10:1:18060; doi:10.1038/s41598-020-75139-w. 33093594 PMC7582924

[34] Empen K, Lorbeer R, Dorr M, Haring R, Nauck M, Glaser S, Association of testosterone levels with endothelial function in men: results from a population-based study. Arterioscler Thromb Vasc Biol. 2012;32:2:481–6; doi:10.1161/ATVBAHA.111.232876. 22095984

[35] Ji H, Zheng W, Wu X, Liu J, Ecelbarger CM, Watkins R, Sex chromosome effects unmasked in angiotensin II-induced hypertension. Hypertension. 2010;55:5:1275–82; doi:10.1161/HYPERTENSIONAHA.109.144949. 20231528 PMC2905778

[36] Vlachopoulos C, Ioakeimidis N, Miner M, Aggelis A, Pietri P, Terentes-Printzios D, Testosterone deficiency: a determinant of aortic stiffness in men. Atherosclerosis. 2014;233:1:278–83; doi:10.1016/j.atherosclerosis.2013.12.010. 24529157

[37] Li J, Chen X, McClusky R, Ruiz-Sundstrom M, Itoh Y, Umar S, The number of X chromosomes influences protection from cardiac ischaemia/reperfusion injury in mice: one X is better than two. Cardiovasc Res. 2014;102:3:375–84; doi:10.1093/cvr/cvu064. 24654234 PMC4030514

[38] Itoh Y, Golden LC, Itoh N, Matsukawa MA, Ren E, Tse V, The X-linked histone demethylase Kdm6a in CD4+ T lymphocytes modulates autoimmunity. J Clin Invest. 2019;129:9:3852–63; doi:10.1172/JCI126250. 31403472 PMC6715385

[39] Chen X, McClusky R, Itoh Y, Reue K, Arnold AP. X and Y chromosome complement influence adiposity and metabolism in mice. Endocrinology. 2013;154:3:1092–104; doi:10.1210/en.2012-2098. 23397033 PMC3578992

[40] Pasquali D, Arcopinto M, Renzullo A, Rotondi M, Accardo G, Salzano A, Cardiovascular abnormalities in Klinefelter syndrome. Int J Cardiol. 2013;168:2:754–9; doi:10.1016/j.ijcard.2012.09.215. 23092857

[41] Chen X, McClusky R, Chen J, Beaven SW, Tontonoz P, Arnold AP, The number of x chromosomes causes sex differences in adiposity in mice. PLoS Genet. 2012;8:5:e1002709; doi:10.1371/journal.pgen.1002709. 22589744 PMC3349739

[42] Chen J, Xu X, Li Y, Li F, Zhang J, Xu Q, Kdm6a suppresses the alternative activation of macrophages and impairs energy expenditure in obesity. Cell Death Differ. 2021;28:5:1688–704; doi:10.1038/s41418-020-00694-8. 33303977 PMC8167088

[43] Arnold AP. Four Core Genotypes and XY* mouse models: Update on impact on SABV research. Neurosci Biobehav Rev. 2020;119:1–8; doi:10.1016/j.neubiorev.2020.09.021. 32980399 PMC7736196

[44] Brull DJ, Murray LJ, Boreham CA, Ralston SH, Montgomery HE, Gallagher AM, Effect of a COL1A1 Sp1 binding site polymorphism on arterial pulse wave velocity: an index of compliance. Hypertension. 2001;38:3:444–8; doi:10.1161/01.hyp.38.3.444. 11566920

[45] Clayton ZS, Rossman MJ, Mahoney SA, Venkatasubramanian R, Maurer GS, Hutton DA, Cellular Senescence Contributes to Large Elastic Artery Stiffening and Endothelial Dysfunction With Aging: Amelioration With Senolytic Treatment. Hypertension. 2023; doi:10.1161/HYPERTENSIONAHA.123.21392. PMC10530538 37593877

[46] Wang Z, Wang DZ, Pipes GC, Olson EN. Myocardin is a master regulator of smooth muscle gene expression. Proc Natl Acad Sci U S A. 2003;100:12:7129–34; doi:10.1073/pnas.1232341100. 12756293 PMC165841

[47] Petsophonsakul P, Furmanik M, Forsythe R, Dweck M, Schurink GW, Natour E, Role of Vascular Smooth Muscle Cell Phenotypic Switching and Calcification in Aortic Aneurysm Formation. Arterioscler Thromb Vasc Biol. 2019;39:7:1351–68; doi:10.1161/ATVBAHA.119.312787. 31144989

[48] Liu R, Jin Y, Tang WH, Qin L, Zhang X, Tellides G, Ten-eleven translocation-2 (TET2) is a master regulator of smooth muscle cell plasticity. Circulation. 2013;128:18:2047–57; doi:10.1161/CIRCULATIONAHA.113.002887. 24077167 PMC3899790

[49] Bank AJ, Kaiser DR. Smooth muscle relaxation: effects on arterial compliance, distensibility, elastic modulus, and pulse wave velocity. Hypertension. 1998;32:2:356–9; doi:10.1161/01.hyp.32.2.356. 9719067

[50] Bussy C, Boutouyrie P, Lacolley P, Challande P, Laurent S. Intrinsic stiffness of the carotid arterial wall material in essential hypertensives. Hypertension. 2000;35:5:1049–54; doi:10.1161/01.hyp.35.5.1049. 10818063

[51] Alsiraj Y, Thatcher SE, Charnigo R, Chen K, Blalock E, Daugherty A, Female Mice With an XY Sex Chromosome Complement Develop Severe Angiotensin II-Induced Abdominal Aortic Aneurysms. Circulation. 2017;135:4:379–91; doi:10.1161/CIRCULATIONAHA.116.023789. 27815372 PMC5470072

[52] Pessoa BS, Slump DE, Ibrahimi K, Grefhorst A, van Veghel R, Garrelds IM, Angiotensin II type 2 receptor- and acetylcholine-mediated relaxation: essential contribution of female sex hormones and chromosomes. Hypertension. 2015;66:2:396–402; doi:10.1161/HYPERTENSIONAHA.115.05303. 26056343

[53] Lincoff AM, Bhasin S, Flevaris P, Mitchell LM, Basaria S, Boden WE, Cardiovascular Safety of Testosterone-Replacement Therapy. N Engl J Med. 2023;389:2:107–17; doi:10.1056/NEJMoa2215025. 37326322

