# Supplementary material for: Testosterone Deficiency Promotes Arterial Stiffening Independent of Sex Chromosome Complement
Source: Res Sq. 2023 Oct 14:rs.3.rs-3370040. Preprint. [Version 1] doi: 10.21203/rs.3.rs-3370040/v1 (PMC10602149; doi:10.21203/rs.3.rs-3370040/v1)
Supplement: Supplement 1 [file NIHPPrs3370040v1-supplement-1.pdf]

Supplementary Files

This is a list of supplementary files associated with this preprint. Click to download.

- SupplementaryFigure1.jpg
- SupplementaryFigure2.jpg
- SupplementaryTable1.jpg
- SupplementaryTable2.jpg
